# Colon Cancer Screening Methods: 2023 Update

**DOI:** 10.7759/cureus.37509

**Published:** 2023-04-12

**Authors:** Maleesha Jayasinghe, Omesh Prathiraja, Dilushini Caldera, Rahul Jena, James Anwar Coffie-Pierre, Minollie Suzanne Silva, Ozair S Siddiqui

**Affiliations:** 1 Medicine, Nanjing Medical University, Nanjing, CHN; 2 Medicine and Surgery, Nanjing Medical University, Nanjing, CHN; 3 Medicine, 37 Military Hospital, Accra, GHA; 4 Neurology/Internal Medicine, Bharati Vidyapeeth Medical College/Bharati Hospital, Pune, IND; 5 Internal Medicine, Nanjing Medical University, Nanjing, CHN; 6 Medicine, GMERS Medical College and Hospital, Dharpur-Patan, Patan, IND

**Keywords:** fobt, fit test, colonoscopy, colorectal cancer, colon, colon polyps, colon cancer surveillance, colon cancer prevention, cancer, colon cancer

## Abstract

Colorectal cancer (CRC) is a significant cause of morbidity and mortality worldwide. National screening guidelines have been implemented to identify and remove precancerous polyps before they become cancer. Routine CRC screening is advised for people with average risk starting at age 45 because it is a common and preventable malignancy. Various screening modalities are currently in use, ranging from stool-based tests (fecal occult blood test (FOBT), fecal immunochemical test (FIT), and FIT-DNA test), radiologic tests (computed tomographic colonography (CTC), double contrast barium enema), and visual endoscopic examinations (flexible sigmoidoscopy (FS), colonoscopy, and colon capsule endoscopy (CCE)) with their varying sensitivity and specificity. Biomarkers also play a vital role in assessing the recurrence of CRC. This review offers a summary of the current screening options, including biomarkers available to detect CRC, highlighting the benefits and challenges encompassing each screening modality.

## Introduction and background

Colorectal cancer (CRC) is the fourth most prevalent cancer, making up 10% of all new cancer cases, and the fifth most prevalent cause of cancer-related death, accounting for approximately 550,000 deaths per year globally. While 25%-50% of CRC patients present at an early stage but later experience recurrence or metastases, 25% of patients are diagnosed at an advanced stage, which is associated with a poor prognosis and a five-year overall survival rate of only 14% [1].

**Figure 1:**
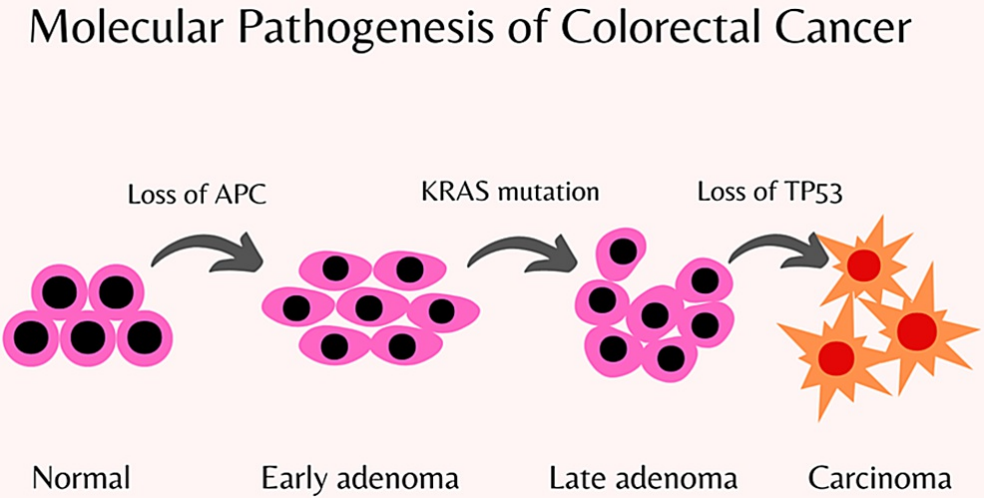
Pathogenesis of colorectal cancer APC: antigen-presenting cell; KRAS: Kirsten rat sarcoma viral oncogene homolog; TP53: tumor protein *p53* gene. Image Credit: Maleesha Jayasinghe

The development of CRC results from alterations in the healthy colonic epithelium, including the development of adenomatous polyps that may multiply and grow in size, causing genetic and epigenetic mutations to accumulate over time (Figure 1).

Those polyps with malignant characteristics have the potential to metastasize, even though not all of them develop into invasive cancer. Due to this unchecked growth, the polyps begin to invade nearby tissues, especially the bowel wall, and eventually travel to distant sites via the lymphatic and circulatory systems [2].

Risk factors for CRC include age, a history of chronic diseases such as inflammatory bowel disease (IBD) and Crohn's disease, a sedentary lifestyle, obesity, poor dietary habits, smoking, and alcohol consumption. Therefore, the increasing prevalence of CRC in industrialized nations can be traced back to an aging population, poor modern dietary habits, and a corresponding rise in risk factors like smoking, insufficient physical activity, and obesity [3].

The treatment of CRC still faces significant clinical challenges in terms of early detection and effective treatment. Finding new tumor-associated molecules that could be used to develop novel clinical diagnostics and therapeutic targets for various malignancies is therefore urgently needed [4].

The goal of CRC screening is to identify early-stage CRC and remove adenomas and sessile serrated lesions (SSLs). Advanced adenomatous polyps can be found using some screening techniques, such as colonoscopy, sigmoidoscopy, computed tomographic colonography (CTC), and, to a lesser extent, stool-based testing, whereas colonoscopy is the best method for finding SSLs. Endoscopic polyp removal lowers the risk of developing CRC and CRC mortality. The “ideal” screening test should be highly sensitive and specific, non-invasive, safe, feasible, and affordable [5-7].

One method for categorizing CRC screening tests is to classify them as either one-step tests (such as colonoscopy, which is both diagnostic and therapeutic) or two-step tests that, if positive, require colonoscopy to complete the screening process. With the exception of colonoscopy, all screening tests involve two steps. The fact that a positive test necessitates a subsequent colonoscopy is a significant drawback of non-colonoscopy-based CRC screening tests such as stool-based, flexible sigmoidoscopy (FS), CTC, or colon capsule endoscopy (CCE). This two-step testing method is more successfully used in organized screening and necessitates strong system-based support to complete the screening cascade. Few select healthcare systems in the United States (US) have organized, programmatic screening, and the majority of screening is done using a one-step method. Individuals who refuse or are unable to undergo a colonoscopy or fecal immunochemical test (FIT), as well as those with incomplete colonoscopies, must undergo one of the additional two-step tests, such as the FS, multitarget stool DNA test (mts-DNA), CTC, or CCE. Studies comparing their effectiveness are scarce. The following section discusses and summarizes the screening options [7].

**Figure 2:**
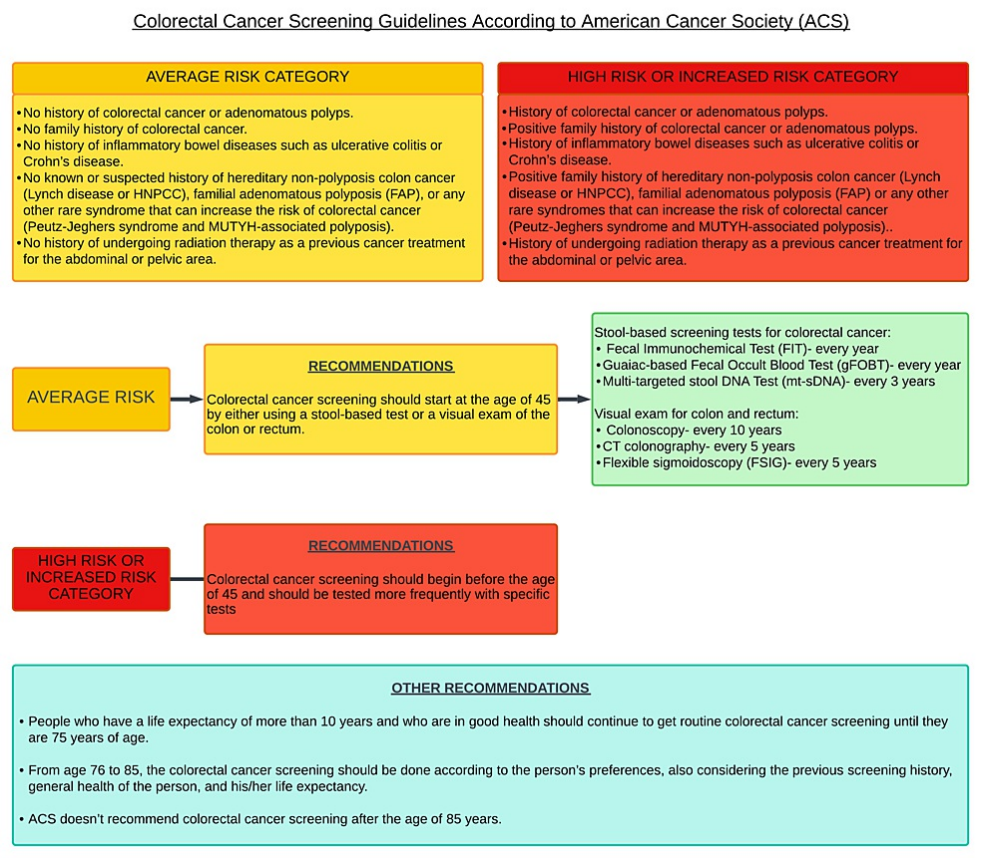
American Cancer Society (ACS) screening guidelines for CRC HNPCC: hereditary non-polyposis colorectal cancer; FAP: familial adenomatous polyposis. Image Credit: Dr. Dilushini Caldera

According to patient preference and test availability, the American Cancer Society (ACS) advises that adults 45 years of age and older with an average risk of CRC undergo routine screening with either a high-sensitivity stool-based test or a visual examination. All positive outcomes on non-colonoscopy screening tests should be promptly followed up with a colonoscopy as part of the screening procedure. The suggestion to start screening at 45 is a constrained suggestion. It is strongly advised that adults 50 years of age and older undergo routine screening [8]. The ACS screening recommendation for CRC is covered in Figure 2.

Preventing and detecting cancer at an early stage are two of the most important benefits of CRC screening. Improving CRC screening uptake and outcomes requires an understanding of the test characteristics, advantages, difficulties, and clinical practice considerations for currently available and emerging CRC modalities. Additionally, further development of cutting-edge screening technologies has the potential to lessen CRC's overall global burden. This article discusses the different CRC screening options currently available, with an emphasis on how early detection is crucial for CRC management.

## Review

**Figure 3:**
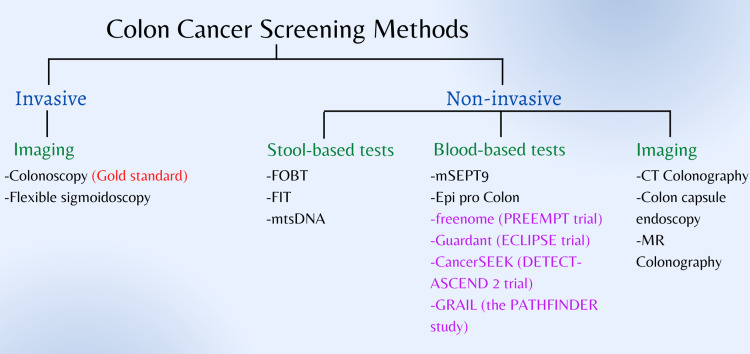
Colon cancer screening methods FOBT: fecal occult blood test; FIT: fecal immunochemical test; mts-DNA: multitarget stool DNA; mSEPT9: methylated Septin9. Image Credit: Maleesha Jayasinghe

Several invasive and noninvasive tests are used to diagnose CRC. Figure 3 depicts all the current, novel, and upcoming screening tests that can be used to detect CRC.

Invasive tests

Colonoscopy for Colon Cancer

The gold standard for CRC screening and adenoma identification is colonoscopy. It is the only screening method that has the ability to combine the removal of an adenoma with therapeutic measures. Currently, colonoscopy is the next step for a definitive diagnosis and potentially curative treatment when other screening methods produce positive results. The terminal ileum, ileocecal valve, and appendiceal orifice are three endoscopic landmarks that help gastrointestinal endoscopists locate the ileocecum during a colonoscopy [9]. CRC and its precursors, lower gastrointestinal hemorrhage, IBD, diarrhea, and therapeutic objectives such as polypectomy, bleeding management, and dilatation of stenotic areas are among the indications for colonoscopy. These disorders are also monitored, diagnosed, and treated using it [10].

The goal of screening is to find CRC at an early stage when it is more likely to be treatable. Colon cancer can be avoided through screening since it can find and remove colorectal polyps before they develop into cancer. These two results can reduce the overall fatality rate from CRC [11]. For patients at normal risk for CRC, the ACS has dropped its recommendation for colonoscopy screening from 50 to 45 years of age. Patients with a personal history of CRC, IBD, polyps, abdominal radiation, a family history of CRC, or those who have a hereditary CRC syndrome must be identified as being at an elevated risk (e.g., hereditary non-polyposis colorectal cancer (HNPCC) or familial adenomatous polyposis (FAP)) [12]. The age distribution of incidence was similar to that of mortality, with those under 75 years old accounting for 37% of cases in 2005 and 42% of cases in 2014 of CRC (men 38%; women 49%). Given that the corresponding number was 26% in 1990 (men, 23%; women, 30%), it is clear that the age of those affected is steadily rising [13]. Colonoscopy with polypectomy is expected to continue to be the cornerstone of CRC prevention, even with new screening tests. No matter if we're performing primary screening, monitoring the results of non-invasive screening tests, surveillance, or symptom analysis, we must make sure that the inspection, lesion characterization, and lesion removal are done with skill [14].

Due to the possibility of luminal recurrence, the required margin of a polypectomy resection is still a subject of significant discussion. In malignant polyps with a margin of excision greater than 1 mm, this varies from 0% to 2%. However, the percentage of relapse varies between 21% and 33% when the resection margin is involved, or when it is less than 1 mm [15].

CRC screening is intended to reduce CRC with the least amount of risk and expense possible. Different outcomes must be measured in order to continuously enhance the quality of colonoscopies. The priority indicators for this subject are the frequency of repeat colonoscopy recommendations made after the procedure, the frequency of adenomas found in average-risk individuals having their first screening colonoscopy, and the frequency of scheduling colonoscopies at appropriate intervals based on current guidelines. Studies show a decrease in CRC mortality when asymptomatic people are screened with sigmoidoscopy and FOBT. However, the best yield for finding polyps is with colonoscopy [16,17].

Although colonoscopy is frequently used as a screening tool in the detection of CRC, its impact on the risks of CRC and related deaths is unknown. The risk of CRC at 10 years was lower among individuals who were asked to have a screening colonoscopy than among those who were assigned to no screening, according to a randomized trial conducted by Bretthauer et al. between 2009 and 2014, comprising presumed healthy men and women aged 55 to 64 who were taken from population registries in Poland, Norway, Sweden, and the Netherlands. Participants in this study were randomly assigned in a 1:2 ratio to either receive an invitation to have a single screening colonoscopy (the invited group) or to receive neither an invitation nor a screening (the usual-care group). The primary objectives were the risks of CRC and fatalities related to them, while the secondary endpoint was death from any cause. A large amount of bleeding occurred in 15 patients following the removal of the polyp. No screening-related deaths or perforations were found within 30 days of the colonoscopy. After a median follow-up of 10 years, 259 incidences of CRC were found in the invited group, while 622 cases were reported in the usual-care group. According to intention-to-screen studies, the risk of CRC at 10 years was 0.98% in the invited group and 1.20% in the usual-care group. The likelihood of dying from CRC was 0.28% in the invited group versus 0.31% in the group receiving conventional care [18].

According to studies, the likelihood of CRC following a colonoscopy is influenced by the quality of the procedure. Schwarz et al. conducted a study in Germany to determine the cumulative incidence of proximal colon CRC in people undergoing colonoscopy based on the physician's polyp detection rate (PDR). The study included people who had a baseline colonoscopy between 2008 and 2017 and classified them according to the procedure at baseline using the German Pharmacoepidemiological Research Database with claims data from 20% of the German population (snare polypectomy, forceps polypectomy, no polypectomy). In each subgroup, persons examined by physicians with a PDR in the lowest quartile were compared with those in higher quartiles and their cumulative CRC incidence during follow-up. Overall, 822,715 persons examined by 1752 physicians were included in the study. One-quarter of the physicians had a PDR of less than or equal to 21.8%. The five-year cumulative CRC incidence was statistically significantly higher in persons examined by physicians with a PDR ≤21.8% compared to >21.8%. It was 69% higher in persons with snare polypectomy, 87% higher in persons with forceps polypectomy, and 48% higher in persons without polypectomy at baseline. Regardless of the baseline results, the study discovered a higher risk of proximal colon CRC in patients seen by physicians with a low PDR, emphasizing the necessity of a high-quality colonoscopy to maximize the preventative impact of colonoscopy on CRC incidence [19].

Over the past few years, local en bloc resection of *pT1 *colon cancer has become more popular. The risk of lymph-node metastases is minimal and does not justify the morbidity and mortality of an oncologic resection in the absence of histological risk factors. Complex benign polyps can be removed using the colonoscopy-assisted laparoscopic wedge resection (CAL-WR) method, which has been proven to be both efficient and secure. It could close the gap for cancers with macroscopic evidence of profound submucosal invasion, giving more patients access to organ-preserving therapies. A recent retrospective study that aimed to assess the radicality and safety of CAL-WR as a local en bloc resection approach for suspected T1 colon cancer investigated the use of CAL-WR for the initial resection of T1 colon cancer. A total of 57 patients who had CAL-WR for probable macroscopic polyps or polyps with biopsy-confirmed high-grade dysplasia or T1 colon cancer were included in the research. A *pT1 *colon cancer was discovered at the pathologic examination following CAL-WR in 27 of these 57 patients. Three cases had histological risk markers for lymph node metastasis, and 70% of those cases had deep submucosal invasion (Sm2/Sm3). An overall R0-resection rate of 88.9% for individuals with *pT1 *colon cancer was reported [20]. 

It has been discovered that colonoscopy with artificial intelligence (AI) assistance improves polyp characterization and detection. But there is a dearth of information from sizable multicenter randomized controlled trials (RCT) in an asymptomatic group. In order to compare AI-assisted colonoscopy with traditional colonoscopy for the diagnosis of adenoma in an asymptomatic population, a multicenter RCT was conducted from November 2019 to August 2021. At six referral centers in Hong Kong, Jilin, Inner Mongolia, Xiamen, and Beijing, asymptomatic individuals between the ages of 45 and 75 were enrolled in a CRC screening program using direct colonoscopy or a FIT. An AI polyp identification system (Eagle-Eye) with real-time alerting on the same monitor as the endoscopic device was employed in the AI-assisted colonoscopy. The overall adenoma detection rate (ADR) was the primary outcome of the study. Secondary outcomes included colonoscopy withdrawal time, the average number of adenomas per colonoscopy, and ADR based on endoscopist experience. Both AI-assisted colonoscopies and standard colonoscopies were randomly assigned to the subjects. Across the two groups, the baseline traits and bowel preparation quality were equivalent. In the AI-assisted colonoscopy, the overall ADR, advanced ADR, ADR of expert and nonexpert endoscopists, and adenomas per colonoscopy were all significantly higher. AI-assisted colonoscopy improved the overall ADR, advanced ADR, and ADR of both expert and nonexpert attending endoscopists in this multicenter RCT in asymptomatic patients [21].

Flexible Sigmoidoscopy

A host of different screening methods have been developed for colon cancer, one of which is FS. FS has gained significant traction in recent years due to convenience. The procedure involves the visual inspection of the descending, rectosigmoid colon and the rectum by the use of a colonoscope after the administration of an enema or laxative to promote distal bowel evacuation [22]. Its merits lie in the fact that it is a simple daycare procedure and the fact that nurse practitioners and non-specialists can be trained to perform it with appropriate training, as well as less recovery time for patients [22]. Randomized trials found that traditional approaches such as FOBT and FS significantly reduced mortality associated with CRC [23-25]. A case-control study by Ko et al. found that colonoscopy as a screening tool had a greater reduction in rates of CRC mortality compared to sigmoidoscopy. This was observed more in the distal colon than the proximal colon [26]. However, a sigmoidoscopy is relatively cheaper, takes less time to perform, has fewer complications, and is more convenient to perform compared to a colonoscopy, which often requires sedation as well. However, FS limits itself to only the distal part of the colon and often requires a colonoscopy to confirm a positive test. Both the ACS and the American College of Gastroenterology (ACG) recommend beginning FS screening at the age of 50 every five years [24]. The 2021 U.S. Preventive Services Task Force (UPSTF) guidelines also recommended the addition of a FIT to FS, which was found to have additional advantages, almost equivalent to the benefits of a colonoscopy in terms of years gained and mortality reduction [27]. FS is often preferred in low-resource settings, in terms of either technology or manpower [28].

Non-invasive tests

Fecal Occult Blood Test

The high-sensitivity guaiac-based FOBT (HSgFOBT) is a stool-based screening technique that uses an oxidation reaction to detect colorectal polyps and malignancies. According to this screening technique, individuals must annually submit three consecutive stool samples, and any abnormal result justifies a colonoscopy to check for colorectal polyps or malignancies. Because colonoscopy and other stool-based screening tests are so widely used in the US, there is not much data on HSgFOBT [29]. FOBT is a feasible method used in the detection of CRC in primary care settings. According to a Saudi Arabian study, although the age-standardized incidence rate was higher than that of the Saudi Cancer Registry, it was still lower than that of other nations. The objective of this study was to assess the performance of FOBT as a CRC screening tool. All individuals over 50 who had completed the FOBT test between January 2002 and March 2017 had their medical data retrospectively examined. 19.7% of the patients who had the FOBT screening received positive results, while CRC accounted for 3.5% of all abnormal pathology reports. The adaptive statistical iterative reconstruction for Saudi Arabia was 26.56 per 100,000 [30]. A meta-analysis of six trials conducted over a 15-year period revealed that screening with FOBT reduced CRC-related mortality but did not lower the incidence of CRC [31].

Fecal Immunochemical Test

FIT evaluates the presence of occult blood in a spontaneous feces sample using an antibody against the globin portion of heme. Notably, the performance of FITs offered in the US varies greatly by brand. Sensitivity for advanced colorectal neoplasia and CRC varies between 25% and 27%, and 74% to 81%, respectively, for two widely used FITs with remarkably consistent performance characteristics [28]. Dietary and medication modifications are not required, and the data are inconclusive as to whether concurrent use of aspirin or antithrombotic medications affects sensitivity, specificity, or positive predictive value (PPV), although the PPV is likely diminished when testing is performed in patients taking these medications [32].

The Danish CRC screening program was initiated in March 2014. To use the FIT, individuals between the ages of 50 and 74 are requested to provide a single stool sample. The initial screening cycle was implemented over a period of four years, but as of 2018, citizens are now invited every two years. A FIT score of 20 g/g feces is deemed positive, and citizens whose FIT test is positive are offered a colonoscopy. If the FIT is negative, the person is asked to provide another stool sample two years later [33]. Nonetheless, a recent study found a strong link between FIT value and risk of interval cancer, even for extremely low values of 10 g/g feces. In low FIT categories, the study revealed that an increase in the screening interval may be feasible. In a two-year screening round of FIT-negative persons with a FIT 10 g/g feces, the overall incidence of interval CRC was 0.07%, with increasing hazard ratios for interval CRC corresponding to increasing FIT values. The total incidence quadrupled to 0.14% when the follow-up was extended by one year to three years, while the hazard ratios remained unchanged. This would imply that for the low FIT categories, a longer screening interval might be safe and appropriate [34].

Despite these encouraging figures, neither adenomas nor more severe diseases are present in 39%-52% of individuals who are referred for FIT-positive diagnostic colonoscopy [35,36]. Two hundred and sixty-eight people had a false positive FIT (FP-FIT), according to a retrospective study by Law et al. on 596 patients who underwent diagnostic colonoscopy after a positive screening FIT. This study showed that hemorrhoids and female sex were linked with higher odds of FP-FIT and fewer advanced adenomas, whereas increasing age and body mass index were associated with lower odds of FP-FIT and more advanced adenomas. In FIT-positive patients, the choice to perform a diagnostic colonoscopy may be influenced by the existence of the above-mentioned indicators [36]. Such FP-FIT results subject patients to unnecessary colonoscopies, which raises the burden and cost of healthcare, subject patients to unnecessary interventions, decrease adherence to treatment with yearly FIT testing and can cause psychological distress for up to six weeks after a typical colonoscopy [35-37].

FIT outcomes are dependent on the grade of CRC, and the location and size of adenomas, despite having good sensitivity and specificity. In a study by Yuan et al., 692 people had their fecal hemoglobin (Hb) level tested using a quantitative FIT (qFIT). The outcomes of the colonoscopy, including the location, size, and histological characteristics of the adenomas, as well as the association between the Hb level, were examined. The qFIT's performance was assessed at various fecal Hb thresholds. Based on the findings of the colonoscopic and pathological investigations, it was determined that 76 patients had advanced colorectal neoplasia (ACRN). Fecal Hb levels were higher in large adenomas (>10 mm) than in small adenomas (10 mm). A greater fecal Hb level was seen in advanced adenomas on the left side of the colon than on the right. Patients with stage III-IV CRC had considerably higher Hb levels than those with stage I-II. The optimal cut-off level for qFIT for ACRN was 400 ng/mL, which had a sensitivity and specificity of 51.3% and 86.4%, respectively. The optimum cut-off level for CRC was 500 ng/mL, and the sensitivity and specificity were 61.0% and 89.1%, respectively [38].

Although FIT has good overall sensitivity for CRC detection, it can miss about one-third of stage I CRCs. In a 2020 study, 435 patients with newly diagnosed CRC had fecal samples taken prior to treatment. The manufacturer's recommended cut-off (17g/g feces) and alternative cut-offs, ranging from 10 to 40 g/g feces, overall and stratified by tumor location, were calculated to determine a FIT's sensitivity for tumors of various T stages and overall TNM stages (according to the Union for International Cancer Control). The FIT detected T1 cancers with 52% sensitivity, T2 tumors with 79% sensitivity, T3 tumors with 93% sensitivity, and T4 tumors with 84% sensitivity at the manufacturer's recommended threshold. The FIT had a sensitivity of 68% for cancers in stage I, 92% for cancers in stage II, 82% for cancers in stage III, and 89% for tumors in stage IV. The FIT detected stage I CRCs with sensitivity values that were 11%-33% lower than for later-stage CRCs and T1 CRCs with sensitivity values that were 22%-52% lower than for tumors of subsequent T stages. With sensitivities of 32% and 52%, the FIT identified T1 and stage I CRCs in the distal colon. Studies are required to improve the noninvasive detection of early-stage CRC because FIT can miss about one-third of stage I CRCs [39].

Studies have revealed that FIT is less effective at detecting colon tumors in the proximal colon. To assess the impact of FIT for CRC screening on overall and site-specific long-term effectiveness, a prospective study of Taiwanese nationwide biennial FIT screening was conducted on 5,417,699 individuals from 2004 to 2009 and was followed up until 2014. Compared to the proximal colon, the distal colon showed greater long-term success, with a 34% significant reduction in advanced-stage CRCs and a 40% reduction in CRC-related deaths. The difference in long-term effectiveness at different sites also sheds light on how to address the low efficacy of FIT screening in the proximal colon [40].

Multitarget Stool DNA Test

Patients can collect a single random stool sample at home for mts-DNA testing without having to make any changes to their diet or medication regimen. The test identifies 10 biomarkers, including altered human DNA and hemoglobin, that are known to be connected to CRC and precancerous lesions. The test creates a composite, single, “negative” or “positive” patient result using the normalizing gene beta-actin and the results from all the biomarkers [41]. The mts-DNA test (Cologuard, Exact Sciences) was approved by the U.S. Food and Drug Administration (FDA). FDA in August 2014 for screening people 50 years of age and older who are at average risk for CRC. In October 2014, the Centers for Medicare and Medicaid Services approved the reimbursement. 

Fecal (or stool) DNA testing is a noninvasive technique that checks for human DNA in the stool, which is primarily shed from the colon. Sensitive assays that target particular genetic and epigenetic biomarkers to distinguish neoplastic lesions from non-neoplastic tissue can detect cells exfoliated by colonic lesions like adenomatous and serrated polyps and cancers that have neoplastically altered DNA. mts-DNA testing, however, carries an unacceptable low PPV to be used as a screening test for CRC and is not intended for users outside of average risk screening, such as individuals with a personal history of polyps, CRC, a family history of polyps or CRC, or risks for genetic diseases such as FAP, Lynch, and IBD [42,43].

The likelihood of discovering an adenoma using an mts-DNA screening strategy is low, and many positive tests are not associated with significant colonoscopy findings. The failure to follow up on a positive test with a colonoscopy is a significant issue that must be taken into account when this screening strategy is implemented. Results of mts-DNA testing in 15 hospitals and 150 outpatient clinics were evaluated in a cohort study by Vakil et al. results revealed that 6,835 mts-DNA tests were conducted between 2017 and 2018. 73% of the 18% of people who tested positive underwent a colonoscopy. Of those who received a positive test result, less than 1% were diagnosed with CRC, 17% had advanced adenomas, 9% had serrated adenomas, and 60% had an adenoma. Five hundred and fifty seven of the 6,835 patients who underwent testing had an adenoma or cancer. Of the 1,242 patients with positive test results, 226 had an advanced adenoma or cancer. Up to 21% of people did not follow through with a colonoscopy after receiving a positive test, and it cost $38,849 to find a single advanced tumor or cancer [44].

In two recent modeling studies, annual FIT and colonoscopies every 10 years were found to be more efficient and less expensive than mts-DNA testing every three years [45,46]. Recent research by Kleinschmidt et al. failed to detect adenomas and colon cancer at a higher rate using mts-DNA than screening colonoscopy in select studies. The study involved a retrospective analysis of patients who had been referred for a colonoscopy to two high-volume outpatient procedural centers due to a positive Cologuard test. In addition to pathologic evidence of CRC, advanced adenomas were also documented. From January 1, 2018 to November 1, 2019, 1,585 patients were examined and referred for a colonoscopy for the international classification of diseases-10 codes R19.5 (other fecal abnormalities) and K92.1 (melena). Of these, 84 were referred for a positive Cologuard test. The findings revealed that 6.4% of people had advanced adenomas and 1.3% had CRC. 68.0% of the patients had hyperplastic polyps or a completely normal colonoscopy. 89.7% of patients had hyperplastic polyps, normal findings, or non-advanced adenomas [45].

Another study discovered that as patients get older, the mts-DNA test's specificity declines. In a study comparing the diagnostic efficacy of the mts-DNA test with FIT alone, 9,989 average-risk individuals who underwent colonoscopy had higher sensitivity for the detection of CRC (92% vs. 74%), advanced adenoma (42% vs. 24%), and SSLs 10 mm (42% vs. 5%), but lower specificity for the detection of CRC or advanced lesions (87% vs. 95%) [7].

It is crucial to remember that other screening tests like colonoscopy and FIT shouldn't be combined with the mts-DNA test. When a person is undergoing other tests at appropriate intervals, there is no need to order or carry out this test [42].

Colon Capsule Endoscopy

The introduction of CCE was a revolutionary screening modality for CRC. The invention of the transistor made it possible to design swallowable electronic radio-telemetry capsules to study the gastrointestinal tract [47]. The year 2000 introduced video-telemetry small-bowel capsule endoscopy (SBCE) for small-bowel investigation. The capsule obtains images while being propelled through the gastrointestinal tract via peristalsis and illuminated by a white-light-emitting diode (LED) [48]. By 2006, Eliakim et al. introduced the first-generation CCE first generation (CCE-1) [48]. CCE-1 had a moderate sensitivity for detecting polyps larger than 6mm [49]. Subsequently, a second-generation CCE (CCE-2) was developed to achieve higher sensitivity [50]. The CCE-2 device provides a higher frame rate, captures images from both ends at a rate of 35 images per second in motion, and has a larger lens angle that offers nearly 360° coverage of the colon [51]. 

CCE is a safe, non-invasive, and effective screening tool to detect CRC and polyps. A recent systematic review conducted by Fanny et al. comprising 2,485 patients concluded that the accuracy of CCE is comparable to that of colonoscopy and superior to that of CT [52]. While colonoscopy currently remains the gold standard tool for CRC surveillance, certain situations may prefer to use CCE as an alternative. Situations that contraindicate the use of colonoscopy or the use of sedation or incomplete colonoscopy highlight the alternative use of CCE as a screening modality. Absolute colonoscopy contraindications in which CCE may be considered are: (1) patient refusal; (2) recent myocardial infarction; (3) hemodynamic instability; (4) peritonitis; (5) recent surgery with colonic anastomosis; and (6) bowel injury or repair [53]. Contraindications to sedation in which CCE may be considered include (1) patients in the early stages of pregnancy; (2) patients who have an allergy to sedatives; and (3) patients who are using contraindicated drugs (such as patients with myasthenia gravis (using diazepam, flunitrazepam), HIV patients on protease inhibitors (e.g., Ritonavir), or patients with acute angle-closure glaucoma (diazepam, flunitrazepam) [54].

Another circumstance that accentuates the use of CCE is during an incomplete colonoscopy. Incomplete colonoscopy occurs due to a range of factors contributing to an incomplete colonoscopy in clinical practice categorized into patient, technical, and operator factors. Patient factors include discomfort, intolerance, body habitus, gender, and age. Technical factors include diverticulosis and ineffective sedation [55]. Results of CCE are also not dependent and do not require an endoscopist or technician's expertise to perform [56]. CCE also does not expose patients to radiation or discomfort from bowel distension. Rapid improvement in the imaging function has led to the performance of second-generation CCE being almost as satisfying as that of colonoscopy [56]. A prospective European multicenter study conducted by Spada et al. showed that the detection rate of ≥6mm and ≥10mm colonic polyps using CCE-2 showed high sensitivity of 84% and 88%, respectively, almost equal to that of such polyps using colonoscopy [57]. Further large-scale studies need to be conducted to confirm that CCE is a highly acceptable modality for screening for CRC.

Adequate bowel preparation is an important factor to ensure successful CCE results [55]. The main limitation of CCE is that it requires a more extensive bowel preparation than colonoscopy or CT colonography because it requires laxatives not only for bowel cleansing but also for promoting the excretion of the capsule, called a “booster” [56]. Bowel preparation for CCE requires a relatively large volume of 2-4 L of liquid laxatives, such as polyethylene glycol (PEG) solution, and several boosters, such as sodium phosphate solution, to ensure intestinal clarity [56]. This is necessary for polyp detection using CCE because CCE cannot aspirate residual feces or inflate the intestinal wall. Proper bowel preparation is vital, as it has a direct correlation with the sensitivity of the CCE results. Sensitivity is higher in patients with good or excellent colon cleanliness than in those with fair or poor colon cleanliness [50].

The extent of bowel preparation remains the largest limitation to the future use of CCE as a screening modality for CRC. Most of the adverse effects reported by the patient are related to colon preparation [50]. Other limitations of CCE include the burden of reading and interpreting the results of the endoscopy and the possibility of overlooking lesions, resulting in missed polyps [56]. The future of CCE use as a screening modality for CRC looks optimistic, with new technologies incorporating artificial intelligence emerging and leading to an automatic diagnosis [58].

CT Colonography

In the last decade, CTC or “virtual colonography” has been used as a novel screening method. The USPSTF guidelines recommend the testing interval be five years, and any abnormal findings should undergo colonoscopy [28]. Its advantages include good safety, ease of performance, and relative safety, although the risk of radiation is currently being debated. Computer-based enhancement techniques are often used in combination for better visualization. It has demonstrated a high degree of accuracy and specificity, especially for polyps over 10 cm. For those polyps smaller than 10 cm, it shows good specificity if the polyps have other suspicious characteristics [59]. The cost of CTCs was found to be less than that of colonoscopies, which is consistent with the findings of the SIGGAR trials, which compared CTC and conventional colonoscopies in 21 national health service hospitals [60]. The disadvantages of CTC are few compared to the risk of perforation and added hospitalization for conventional colonoscopies. The risk of radiation exposure from paired CTC scans was estimated to be around 0.14% for 50-year-olds and almost half in 70-year-old patients, with a significant benefit-risk ratio [61].

MRI Colonography

Owing to the risks associated with CTCs, magnetic resonance colonography (MRC) began being evaluated as a diagnostic modality for CRCs. They are free of radiation and can be enhanced by the use of intravenous contrast for evaluation of the entire abdomen. MRC was shown to have low sensitivity and specificity, especially for small polyps and in individuals with average risk [62,63]. Adenoma detection rate is considered to be the most important aspect of any diagnostic modality and was found to be low for MRC, especially for lesions below 5 cm [62,64]. This was due to limited spatial resolution. However, since these lesions are unlikely to have malignant potential, it does not limit their use for screening compared to conventional colonoscopies. It is a well-tolerated procedure without anesthesia or sedation [65]. Techniques, such as dark-lumen microscopy, were used to improve the reliability of MRC by detecting colonic inflammation better and leaving out false positives [66,67]. Therefore, screening with colonoscopies remains the gold standard for detecting CRCs, barring all the procedure-associated complications, but MRCs represent an attractive alternative to conventional colonoscopies.

Colon cancer biomarkers

Currently, the concept of biomarkers, or biological markers, is more fluid and is continually being updated. According to the current clinical standards, measuring the biomarkers for CRC should be cheap and easy to identify cancer, should be able to find out the prognosis of the disease, and should predict the response to a certain treatment, which subsequently improves the patient’s prognosis and quality of life. During the early stages of colorectal carcinogenesis, epigenetic modifications tend to occur more often than genetic mutations, indicating that they may be more useful as the next generation of diagnostic biomarkers for the identification of colonic polyps and malignancies [68].

Aberrant DNA methylation of the promoter region is a critical mechanism for the deactivation of tumor-suppressing genes. By using this mechanism, it is possible to mute genes that are involved in each stage of tumor development. This epigenetic event can be reversed by DNA methylation inhibitors [69]. This aberrant methylated DNA can act as biomarkers in tumor tissues and body fluids, thus helping to detect cancer early [69-71].

Stool-Based Biomarkers

Identification of CRC in stool samples can be done by the MethyLight analysis of fecal DNA, which showed secreted frizzled-related protein gene 2 (SFRP2) methylation as a sensitive single DNA-based biomarker [72]. Although the specificity of the SFRP2 methylation is high for CRC as well as colorectal adenoma, it is found to have moderate to low sensitivity. But compared to the FOBT, the accuracy of fecal DNA methylation biomarkers was high [73]. Furthermore, patients with precancerous lesions could also be detected by the aberrant methylated SFRP2 [74].

The vimentin gene (*VIM*) is usually not expressed in normal colon cells. Aberrant DNA methylation of the Vimentin exon 1 has been particularly seen in cancers arising from the proximal colon, with a specificity of 90% [75].

Aberrant DNA methylation of a potential tumor suppressor gene called Tissue Factor Pathway Inhibitor-2 (*TFPI-2*), which belongs to a group of embryonic cells called the Polycomb group (PcG)-marked genes, can be detected early in colorectal carcinogenesis [76].

Other noninvasive DNA-based hypermethylated genes that can be found in stool samples are GATA binding protein 4, GATA binding protein 4​​​​​​5, Syndecan-2 (SDC2), Methylguanine Methyltransferase (MGMT), Cyclin-Dependent Kinase Inhibitor-2A (CDKN2A), MutL homolog 1 (*MLH1*), and *N-Myc *downstream-regulated gene-4 (NDRG-4) [77-80].

Comparing the FIT with a noninvasive, mts-DNA test that includes quantitative molecular assays for Kirsten rat sarcoma viral oncogene homolog (*KRAS*) mutations, aberrant N-Myc downstream-regulated gene 4 (*NDRG4*), bone morphogenetic protein 3 (*BMP3*)* *methylation, and β-actin, plus Hb immunoassays, the mts-DNA test detected more cancers than the FIT, but it has shown a significant number of false positive results. When compared to the FIT, the mts-DNA has a sensitivity of 93% for stages I-III CRC, and the sensitivity of the test increased with lesion size and grade of the adenoma and sessile serrated polyps [81].

Blood-Based Biomarkers

A study that was done to identify the epigenetic molecular markers in plasma to detect CRC early has shown that tumor-specific methylation of adenomatous polyposis coli (APC), O‐6‐methylguanine‐DNA methyltransferase (*MGMT*), *RASSF2A,* and Wet inhibitory factor 1​​​​​​​ (*Wif-1*)genes could be valuable biomarkers. The above-mentioned genes are methylated in colorectal adenoma. According to recent studies, abnormal methylation of the tumor suppressor gene in plasma or blood in CRC patients may serve as a substitute marker for monitoring CRC [82].

As the best-performing marker panel, the combination of Septin 9 (SEPT9) and nerve growth factor receptor (NGFR) has been discovered, as they both have good sensitivities and specificities. But in CRC samples, SEPT9 has a higher methylation, which gives it the upper hand in being a successful colorectal screening marker in the blood [83]. In comparison with the FIT, SEPT9 was shown to have better sensitivity and specificity for detecting CRC, but on the downside, both the FIT and SEPT9 had lower sensitivity for detecting advanced adenomas [84].

Biomarkers for Prognosis

APC: *APC* is a tumor suppressor gene, and the inactivation of APC due to somatic mutations causes colorectal adenomas and cancers. MicroRNA 21​​​​​​​ (*MiR-21)* activates the Wnt pathway when *APC* is inactivated. Thus,* MiR-21* overexpression in CRCs has been shown to have poor survival and additionally gives information about the prognosis of advanced disease [85].

Chromosome 18q allelic imbalance or loss of expression: CRCs with chromosome 18q allelic imbalance or loss of expression have been shown to have a poor prognosis, but more studies are needed to understand its role in stage II-III cancers [86].

*MLH1* methylation: Women with HNPCC who develop endometrial cancer have been shown to have microsatellite instability (MSI). In primary colon cancers, methylation of the* MLH1 *promoter region is a subset of *MSI*. *MLH1* inactivation in an early stage of primary CRC has been shown to be an important biomarker in advanced diseases, thus having a good prognostic value [87].

Vascular endothelial growth factor (VEGF): VEGF is expressed in CRC, compared to minimal expression of VEGF in the normal colon mucosa, thus acting as a prognostic marker [88].

*P53* mutations: Mutations of the tumor suppressor gene* P53* is an early event of ulcerative colitis-associated carcinogenesis, in which the mutation of the *P53 *gene is preceded by the loss of heterozygosity in the development of colon cancer, showing a poor prognosis [89].

Insulin-like growth factor-II mRNA-binding protein 3 (IMP3): IMP3 is expressed in a variety of solid tumors and fetal tissues. Although IMP3 is expressed in the cancer lesions, it was barely detectable in the tissue equivalents nearby. When compared to non-metastatic CRCs, IMP3 was shown to be strongly expressed in CRCs with lymphoid metastases. Therefore, IMP3 is commonly found in late-stage metastatic CRCs with a poor prognosis [90].

Suppressor of mothers against decapentaplegic​​​​​​​ 4 (*SMAD4)*: The mutation of *SMAD4* is an important genetic change that happens in CRCs, where SMAD4 protein expression is lost due to the mutation of this gene. *SMAD4*, which is located on chromosome 18q21, has been associated with a poor prognosis in CRC [91]. 

Biomarkers for Diagnosis

HNPCC: HNPCC is autosomal dominant and is caused by germline mutations in the mismatch repair genes MutS homolog (*MSH*)* 2*, *MSH6*, *MLH1*, and postmeiotic segregation increased 2​​​​​​​* (PMS2*). Therefore, it helps in the early detection of CRC [92,93].

Telomerase: Telomerase maintains the integrity of the telomeres, which are repetitive DNA sequences that are located at the ends of the chromosomes and protect the DNA from degradation. Telomerase activity and telomere status (length and T/N ratio) serve as diagnostic and predictive factors for CRCs [94].

Insulin-like growth factor binding protein 2 (IGFBP2) and Insulin-like Growth Factor II (IGF2): IGFBP2 and IGF2 levels are increased in the plasma of CRC patients. Therefore, IGFBP-2 is considered a diagnostic and prognostic factor for CRCs, while plasma IGF-2 levels are predictive of the survival of CRCs [95].

Limitations

This analysis depends on a survey of open-access research journals published between 2007 and 2023; hence, we might have missed crucial information from full-text journals with a subscription fee and research articles from before 2007. Additionally, we might have missed studies published in other languages because our investigation only included studies written in English.

## Conclusions

CRC is a major cause of mortality around the world. Two of the most significant advantages of CRC screening are cancer prevention and early cancer detection. Understanding the benefits, challenges, and clinical practice considerations of CRC screening modalities is necessary to improve CRC screening uptake and results. This study provides an overview of the different screening modalities, including biomarkers for CRC detection, while highlighting the advantages and drawbacks of each screening technique.
